# The Care of the Eyes*Read before the Chicago Dental Society, November 19, 1918.

**Published:** 1919-02

**Authors:** Cassius D. Wescott

**Affiliations:** Chicago, Ill.


### THE CARE OF THE EYES *

BY CASSIUS D. WESCOTT, M.D., CHICAGO, ILL.

The eyelids are for the protection of the globe, their frequent movements, in winking, for polishing the cornea which is the window of the eye, and the first lens of the camera.

The skin of the lids is the most delicate on the surface of the body, the most easily irritated, and inflamed. Along the edges of the lids are many hair follicles and oil glands, which are easily infected.

An infected hair follicle is, as you know, a stye, and an infected meibomian, or oil gland, is called a chalazion. As we usually see these things they are regarded as trifling, but in a professional man, doing such work as you and I do, they are of real moment, for they are a bar to full efficiency, to say nothing of the pain and discomfort which they cause. Undoubtedly there are constitutional conditions which predispose to these things, but eye strain and direct infection have much to do with their causation.

I have seen chancre, sporotrichosis, blastomycosis, and tuberculosis of the lids, all due to handling the lids, or rubbing the eye with unclean fingers. We should be very careful not to touch the eyelids without washing the hands, and at least twice a day, we should bathe them with warm water and a good soap, followed by a dash of cold water.

If the delicate, mucous membrane covering the globe and lining the lids, becomes irritated by dust or smoke or sulphur gases which are so abundant in our atmosphere just now, there is an increased sensitiveness which

* Read before the Chicago Dental Society, November 19, 1918.

is aggravated by reading or other near work, and there is an increased secretion of mucous, which causes an annoying blurring of vision, both conditions tending to reduce ocular efficiency.

Cold bathing of the closed lids, two or three times a day, has a tonic effect upon the circulation of the eye, but we should not wash the eyes out with plain water. It is best to thicken it a little with boric acid, common salt, or borax—an isotonic solution is much more agreeable to the eye, and just as cleansing as water. If the eyes are persistently red, or irritable, more astringent lotions may be desirable.

Hot water should not be used except upon the advice of an oculist. While very useful in some acute inflammations of the eye ball, both external and internal, hot water is too relaxing to be used frequently under other conditions. The use of a simple cleansing lotion, once or twice a day regularly, could do no harm, and would be very helpful to most city dwellers.

The commonest accident to the human body is the getting of a mote into the eye, and the delicate structures of the globe are so easily infected, and so often permanently damaged by slight injury, that everyone should know how to examine an eye for a foreign body, and to remove one, if present, if it can be done without the skill and appliances possessed only by the eye surgeon. A wooden toothpick, upon the end of which a little clean cotton wool has been tightly twisted, is a very effective instrument for turning a lid, and removing a mote from beneath it, and many foreign bodies lodged in the cornea may easily be removed by the same means if a drop of four per cent. cocain solution is first installed into the eye. An eye containing a foreign body which can not easily be removed, or an eye which has been cut, or scratched, should be tied up immediately with a sterile dressing, or a clean handkerchief, until an expert can be consulted. All penetrating wounds of the eye ball, and even superficial wounds of the cornea, should be regarded as serious, because if they do not heal promptly, and without infection, sight may be destroyed, or greatly impaired. Immediate skilled attention is demanded and can not be of too high a quality.

There are various inflammations of the eye, both from external and autogenous infections. Some are serious and others self-limited and simply disabling. All call for early diagnosis and skillful treatment, and sometimes more can be done to avoid disaster in the first day than in the rest of the patient's lifetime. Focal infections, especially of the teeth and sinuses, play a very important role in eye surgery. Trifling injuries of the eye often result disastrously when complicated by autogenous infection, and many intraocular inflammations are now known to be due to bacteria.

The most widespread of all eye troubles is eye strain. Eye strain and its effects cause more pain and disability than all other eye troubles put together. It probably is responsible for more headache than all other things, to say nothing of eye pain and fatigue, facial spasm, nervous dyspepsia, nausea, vertigo, general fatigue and nervousness, lack of power of concentration, sleeplessness and many other ills, aside from its effect upon the health of the eye itself. As we ordinarily consider it, eye strain is due to our efforts to use our imperfect eyes without correcting glasses, or with glasses improperly fitted, but a perfect eye may be strained by improper use, by too much work without proper periods of rest, and in bad light. But of course it is the defective eyes that cause the most trouble, and there are very few eyes in which we find no measurable optical defect.

Only about ten per cent. of all eyes conform so closely to type that we call them normal. At least sixty per cent. are so defective as to cause ill health, reduced efficiency, or poor sight. The defects of the balance are compensated for by a vigorous accommodation in strong, or insensitive individuals, mostly in out-of-door occupations.

We are all born hyperopic, and the new born baby sees nothing distinctly. He often manifests little muscular co-ordination too, and alarms his mother by looking cross-eyed. If the baby is well nourished, and escapes the serious diseases of infancy, the eyes grow and round out with the development of the body generally. Many children of school age, however, have very defective eyes, and as soon as they are more confined in a schoolroom, which, perhaps, is not too well lightened, and begin to use the eyes more at the reading distance they may begin to have red eyes and to complain of headaches, or other symptoms of eye strain. We see many hyperopic eyes improve when glasses are worn, and remain healthy and strong with eye strain relieved. When errors of refraction are not corrected early, the tendency is toward the development of myopia, or near sight, especially if there is much astigmatism and the eyes are improperly used, in bad light, bad position, etc., but headache, restlessness, nervousness, or apparent dullness and stupidity are usually manifested first. The backward child is usually a defective child and easily becomes a truant.

Astigmatism is that condition of the eye in which the refraction is not the same in all meridians. It is usually due to imperfect curvature of the cornea. Obviously we may have a great variety—hyperopic, myopic, simple, compound, and mixed. It is usually a congenital defect and seventy-five per cent. of all eyes are more or less astigmatic. In high degrees of astigmatism it is impossible for the patient to improve his sight by any effort and he ceases to try, contenting himself with his poor vision and losing his symptoms of strains, but in the low and moderate degrees it is possible, as in hyperopia, to improve the sight by an unconscious effort and symptoms of eye strain are the result. Some of the very worst headaches that I have seen have been in indoor workers, with perfect vision, but who saw well at the expense of a constant effort. When the necessity for the excessive use of the accommodation was relieved by glasses the headache ceased promptly. *Eye strain is not inconsistent with perfect vision.*

One of the most prolific sources of eye strain is faulty illumination. The eye "reacts" most easily, in steady diffuse day light, such as we get in our offices for a few hours on clear days, from a north window. Few of us have day light long enough and sufficiently uniform for our work. We must therefore have artificial light, and the problem is to get that form of illumination which will make for the greatest visual efficiency. If we have too little light of any kind, we will strain our eyes, because in order to see things well enough we must get them very near in order to get a larger retinal image than would be necessary in a stronger light. As the amount of accommodation, and convergence used are directly dependent upon the nearness of the object, the nearer we are obliged to approach the thing we wish to see, the more effort we will expend in order to see it.

While the illuminating engineers tell us that good diffuse day light, from a north window, measures, photometrically, about two foot candles, there is a great variation in the amount of light required by different people, and at different ages in order to see most easily. Generally speaking, the older we grow, the more light we require, but we should always remember that more harm comes from glare and too much light, than from not enough, and should always strive to obtain a soft indirect light which gives us clear vision with the least discomfort.

From the foregoing it is evident that the chief problem in the care of our eyes is to avoid eye strain. Every child should have a careful physical examination before beginning school work in order to discover any defects. Those which can be remedied should be corrected and if there are some which can not be helped, his life should be so adjusted as to enable him to accomplish most with the least wear and tear. We all dislike to see children wear glasses, and no doubt the constant use of spectacles interfere somewhat with a boy's normal activities, but very often can be accomplished by the use of glasses for near work only, and it is seldom necessary to wear them all of the time to the exclusion of such games as football, etc.

As I have already hinted, the early correction with glasses of hyperopia and astigmatism often prevents the development of myopia with its serious consequences, and my own records show that the early use of glasses often promotes the normal growth of the eye, and the spectacles may frequently be discarded after a few years. Sometimes, indeed, they are of use to bridge over a period of special stress and may be discarded after a few months, or a year. Glasses that are needed and which are properly fitted, can do no harm, but only good. If they are not needed, or are improperly fitted, they will usually be rejected and not worn.

Physiological changes are occurring constantly, and the eyes should be re-examined from time to time, and the lenses readjusted to meet the changing needs of the eyes. All need glasses for near work at above forty-five, even if the eyes are quite normal, because of the changes due to age; and after that time the eyes should be thoroughly examined every two years. The question of the constant use of glasses at any age is a matter of the individual, and must be decided after due consideration of the condition of the eye, the nature of one's work, etc. The adjustment of the lenses before the eyes is a matter of great import, because of the fact that we get the true effect of a lens only when we look through its center at right angles to its plane. A lens for the correction of astigmatism has an axis which always must be parallel to the best meridian of the eye. Spectacles are best for the correction of astigmatism because the correct adjustment is more easily maintained. If astigmatism is not accurately, and constantly corrected, the relief of eye strain is not only incomplete but the strain may be aggravated.

Glare and excessive light cause irritation and congestion of the choroid and retina predisposing the eye to inflammation of these delicate structures with resulting malnutrition of the lens, and the production of cataract. Indirectly there is much fatigue from retinal exhaustion and pupillary spasm. The remedy, it seems to me, is indirect illumination with light walls, preferably cream color or light gray, and a sufficient number of units to enable us to regulate the intensity of the diffused light. Of course such a plan is expensive, as compared with direct lighting, but the saving in comfort and sight will make it pay.

If the indirect method is not available, for any reason, we should adopt a plan which will at all times protect our eyes from all direct rays and which is susceptible of variation to suit the conditions. In my operating on the eye ball, I use the Nernst lamp which gives all the light required and can be so adjusted as to position and distance that I can secure just the amount of light needed without direct light in my own eyes. Whether this lamp would be practical in your work I do not know.

I must, however, warn you against any form of illumination which exposes your eyes to direct rays, especially from an electric lamp. Day light should be used as far as possible, and it seems to me that a perfectly diffused, indirect light would be the best substitute. Glasses should be used for the full correction of presbyopia, and for all errors of refraction before the presbyopic age. The form of the glasses is a matter of individual choice. Frequent rests, or change of focus, during near work, are very necessary in order to avoid strain.

The general directions which I have suggested are of course applicable to the dentist as well as to all who use the eyes much for careful, painstaking work.—Abstract from *Journal National Dental Association.*

